# Acute exposure of mice to high-dose ultrafine carbon black decreases susceptibility to pneumococcal pneumonia

**DOI:** 10.1186/1743-8977-7-30

**Published:** 2010-10-19

**Authors:** Ananth Tellabati, Vitor E Fernandes, Friederike Teichert, Rajinder Singh, Jamie Rylance, Stephen Gordon, Peter W Andrew, Jonathan Grigg

**Affiliations:** 1 Department of Infection Immunity and Inflammation, University of Leicester, Leicester, UK; 2 Cancer Biomarkers and Prevention Group, Department of Cancer Studies, University of Leicester, LE1 7RH, UK; 3 Liverpool School of Tropical Medicine, Liverpool L3 5QA, UK; 4 Centre for Paediatrics, Blizard Institute of Cell and Molecular Science, Barts and the London School of Medicine and Dentistry, Queen Mary University London, London, E1 2AT, UK

## Abstract

**Background:**

Epidemiological studies suggest that inhalation of carbonaceous particulate matter from biomass combustion increases susceptibility to bacterial pneumonia. *In vitro *studies report that phagocytosis of carbon black by alveolar macrophages (AM) impairs killing of *Streptococcus pneumoniae*. We have previously reported high levels of black carbon in AM from biomass smoke-exposed children and adults. We therefore aimed to use a mouse model to test the hypothesis that high levels of carbon loading of AM *in vivo *increases susceptibility to pneumococcal pneumonia.

**Methods:**

Female outbred mice were treated with either intranasal phosphate buffered saline (PBS) or ultrafine carbon black (UF-CB in PBS; 500 μg on day 1 and day 4), and then infected with *S. pneumoniae *strain D39 on day 5. Survival was assessed over 72 h. The effect of UF-CB on AM carbon loading, airway inflammation, and a urinary marker of pulmonary oxidative stress was assessed in uninfected animals.

**Results:**

Instillation of UF-CB in mice resulted a pattern of AM carbon loading similar to that of biomass-smoke exposed humans. In uninfected animals, UF-CB treated animals had increased urinary 8-oxodG (P = 0.055), and an increased airway neutrophil differential count (P < 0.01). All PBS-treated mice died within 72 h after infection with S*. pneumoniae*, whereas morbidity and mortality after infection was reduced in UF-CB treated animals (median survival 48 h vs. 30 h, P < 0.001). At 24 hr post-infection, UF-CB treated mice had lower lung and the blood S*. pneumoniae *colony forming unit counts, and lower airway levels of keratinocyte-derived chemokine/growth-related oncogene (KC/GRO), and interferon gamma.

**Conclusion:**

Acute high level loading of AM with ultrafine carbon black particles *per se *does not increase the susceptibility of mice to pneumococcal infection *in vivo*.

## Background

*Streptococcus pneumoniae *is responsible for a significant proportion of global pneumonia deaths [1]. Growing antibiotic resistance, and lack of availability of antibiotics and vaccines, has focussed attention on the preventable environmental risk factors for bacterial pneumonia. In low-income countries, the burning of biomass fuels results in concentrations of inhalable carbonaceous particulate matter (PM) exceeding 8000 μg/m^3^, [2] with young children and women differentially exposed [3],

Epidemiological studies strongly suggest that indoor PM from biomass and solid fuel combustion is one of the most significant environmental risk factors for bacterial pneumonia in low-income countries [4]. However to date, little is known about how carbonaceous PM increases susceptibility to pneumococcal infection. An important component of pulmonary defences against pneumococcal pneumonia is the alveolar macrophage (AM) [5]). Studies of AM *in vitro *suggest that phagocytosis of PM impairs the ability of AM to subsequently kill bacteria. First, Lundborg *et al *[6] reported a dose-dependent increase in the survival of *S pneumoniae *in rat AM loaded with ultrafine carbon black *in vitro*. Second, Zhou and Kobzik [7] reported in a mouse model, that AM phagocytosis of carbonaceous urban PM *in vitro *impairs their ability to subsequently internalise *S. pneumoniae*.

We have previously reported high levels of carbon in AM associated from biomass-smoke exposed children and women living in Ethiopia [8], and from adults living in Malawi [9]. Since elemental carbon nanoparticles are a major, and potentially toxic, component of biomass smoke PM [10], we hypothesised that susceptibility to pneumococcal pneumonia is increased by carbon loading of AM *in vitro*. Using a mouse model, we sought to test this hypothesis by loading AM with ultrafine carbon black (UF-CB) *in vivo *(2× doses of 500 μg UF-CB), and then assessing morbidity and mortality to subsequent intranasal pneumococcal infection.

## Results

### Ultrafine carbon black loading

**Figure 1:**
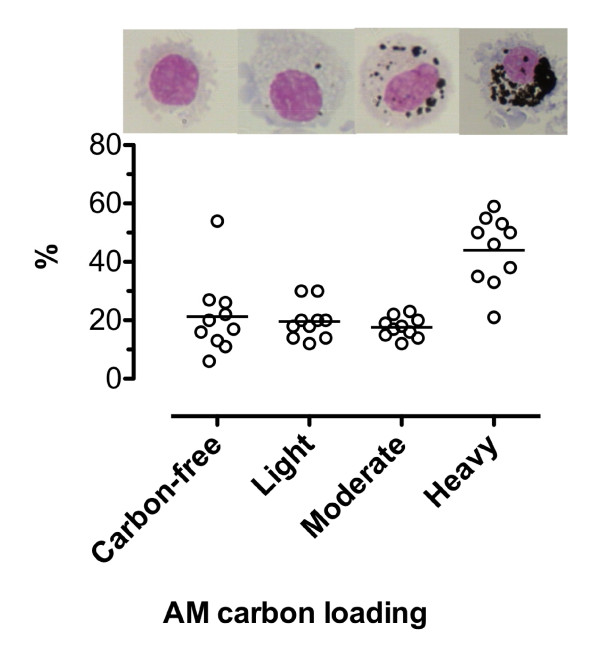
**Carbon loading of alveolar macrophages (AM) from uninfected mice exposed to 2× doses of 500 μg ultrafine-carbon black (UF-CB) in 50 μL phosphate buffered saline**. Bronchoalveolar lavage was performed 72 h after the second dose on day 4. Carbon loading was assessed semi-quantitatively as described in Methods. Data are from 10 animals. Bar represents mean. Representative images of BAL fluid AM loading are shown in the upper panel.

**Figure 2:**
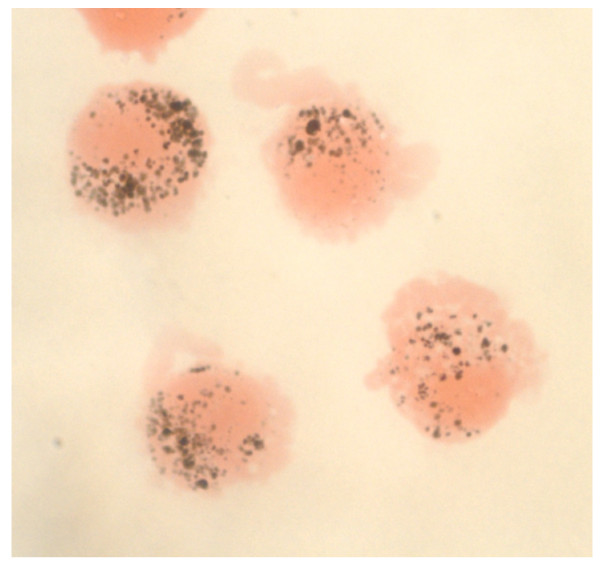
**Carbon loading of human alveolar macrophages (AM) from an adult exposed to biomass smoke in Malawi**. AM were obtained by bronchoalveolar lavage. Image taken under oil immersion (x100). Black material represents phagocytised elemental carbon and the pattern of loading is compatible with moderate- to heavy-loading of AM in UF-CB exposed mice (Figure 1).

The effect of UF-CB alone was assessed in uninfected animals by bronchoalveolar lavage (BAL) and lung histology. No short- or long-term morbidity was observed in animals exposed to UF-CB alone. Bronchoalveolar lavage of PBS-alone treated animals showed no evidence of AM carbon (n = 10). In UF-CB alone treated animals (n = 10), 44 ± 1.1% of AM were heavily-laden with carbon and 17.6 ± 1.1 moderately-laden (Figure 1). The pattern of carbon in mouse AM after instillation of UF-CB was similar to that seen in AM from biomass-smoke exposed humans (Figure 2) [8,9].

**Figure 3:**
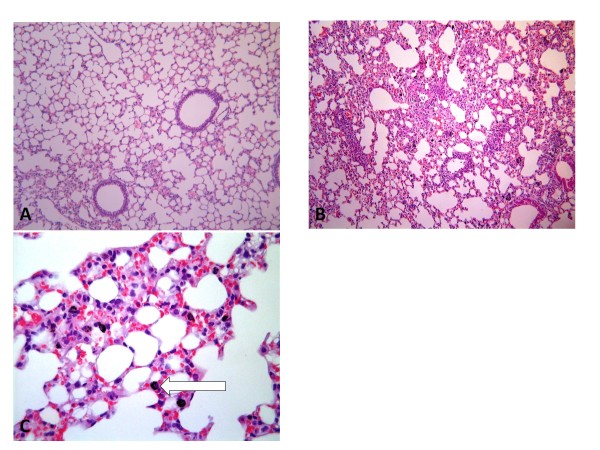
**A,B,C; Lung histology of uninfected mice exposed to**; **A**) intranasal PBS and **B**) 2× doses of 500 μg ultrafine-carbon black (UF-CB). Sections were imaged under light microscopy (200×). Figure **C**) shows lung tissue of an UF-CB exposed animal under higher power (400×). Changes induced by UF-CB are given in Table 1. The most distinctive feature is carbon-laden alveolar macrophages (arrow). There is no evidence of free carbon in the airway, or significant uptake of carbon by other airway cells.

**Figure 4:**
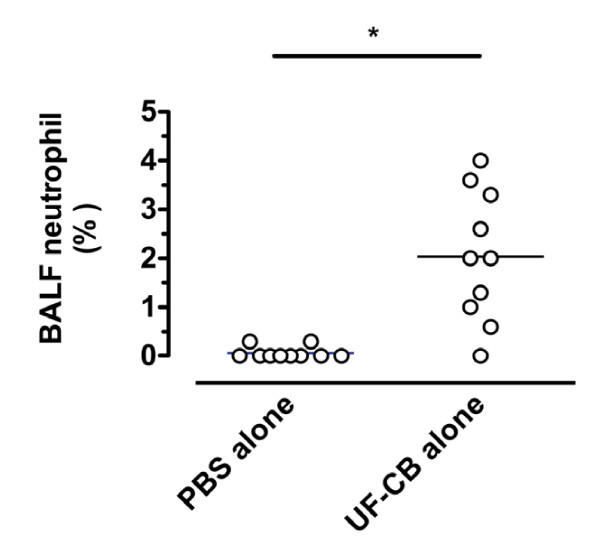
**Neutrophil differential count in bronchoalveolar lavage fluid mice receiving either 2× doses on intranasal sterile PBS alone, or 2 doses of 500 μg ultrafine-carbon black in 50 μL PBS alone (UF-CB)**. BAL was done 72 h after the second intranasal dose. Each symbol represents an individual mouse. Bar represents mean, n = 10, *P < 0.01 by unpaired t test.

**Figure 5:**
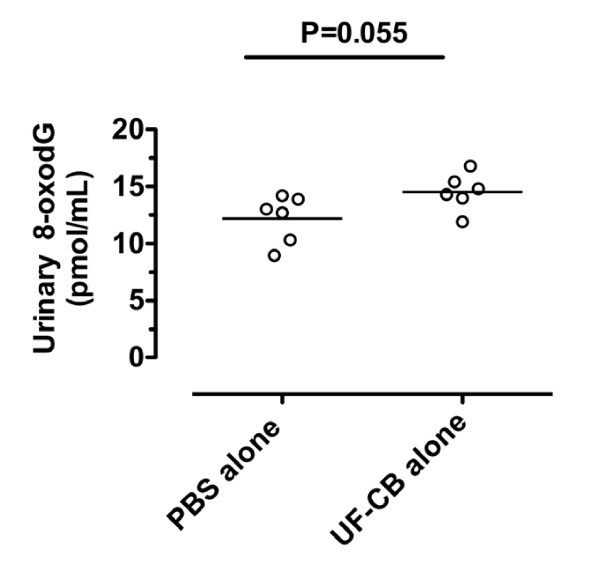
**Urinary 8-oxodG in the urine of mice treated with i) 2× intranasal doses of PBS alone, and ii) 2× intranasal doses of 500 μg ultrafine-carbon black (UF-CB) in PBS alone**. Urine was collected 72 h after the second intranasal instillation. 8-oxodG was assayed by liquid chromatography-electrospray ionization-tandem mass spectrometry and results corrected for specific gravity. Bar represents mean, n = 6 per group, * *P *= 0.055 by unpaired t test.

Lung histology confirmed no inflammation and no AM carbon in mice exposed to PBS alone (Table 1, Fig 3A). After UF-CB alone, most carbon was in AM (Table 1, Fig 3B,C), although minimal uptake of carbon by bronchiolar epithelial cells was also observed (Table 1). In UF-CB alone treated animals, there was a mild inflammatory response characterised by infiltration of leukocytes, predominantly neutrophils (Table 1). Compatible with histological findings, analysis of BAL fluid at 72 h post instillation showed an increased proportion of neutrophils in UF-CB alone treated animals (n = 10, 0.06 ± 0.04% vs. 2.0 ± 0.42%, *P *< 0.01, Fig 4). UF-CB alone treated animals had increased urinary 8-oxodG (12.1 ± 0.8 vs. 14.5 ± 0.6 pmol/mL, *P *= 0.055, vs. PBS) suggestive of increased pulmonary oxidative stress (Fig 5).

**Table 1:** Lung histology in uninfected mouse lung tissue 72 h after intranasal instillation *in vivo *of either phosphate buffered saline (PBS) or ultrafine carbon black (2 × 500 μg).

Alveolar		Bronchiolar	
PBS	Ultrafine-Carbon black	PBS	Ultrafine Carbon Black
Minimal diffuse alveolar microvascular congestion	Moderate to marked black particle inclusions - mainly within alveolar macrophages associated with moderate to marked secondary pneumonic reaction	Normal	Moderate sporadic black particle inclusions within bronchiolar epithelium
Minimal sporadic alveolar macrophage and neutrophil foci	Marked diffuse alveolar microvascular congestion		Marked multi-focal leucocyte infiltration of airway walls - mainly neutrophils and mononuclear cells
	Marked diffuse pneumonitis with consolidated microvascular leak		Moderate multi-focal hypertrophy of transitional airway epithelium
			Moderate to marked focal hypertrophy of mesothelium

### Survival

**Figure 6:**
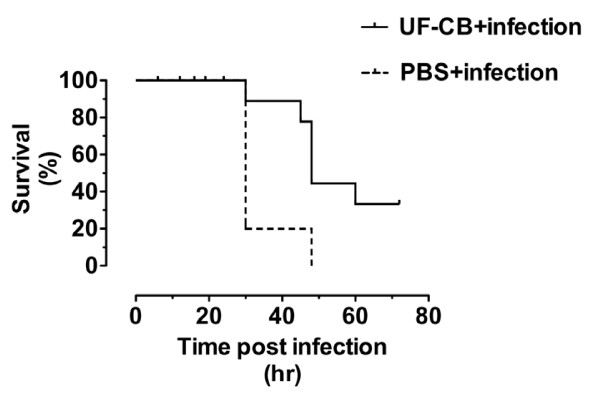
**Survival curves of mice infected with pneumococci and pre-treated with; i) 2× intranasal doses of sterile PBS (PBS +infection) and ii) 2× intranasal doses of 500 μg ultrafine-carbon black (UF-CB + infection)**. Animals treated with PBS alone or UF-CB alone had no morbidity (data not shown). Data shown are representative of >4 separate experiments done at different times each with 10 animals per group. In the data plotted, median survival of PBS treated mice was 30 h. In contrast, median survival in UF-CB treated mice was 48 h (hazard ratio 8.5 (95% CI 2.0 to 36), *P *< 0.001 by log rank test).

Survival after infection with *S. pneumoniae *was assessed up to 72 h. Initial experiments included uninfected (PBS alone and UF-CB alone) animals. However, no morbidly or mortality was observed in these uninfected animals. Additional survival experiments therefore used infected mice only (Figure 6). All PBS-treated animals succumbed from pneumococcal infection by 72 h. In contrast, exposure to UF-CB consistently delayed time to death from infection and increased morbidity-free survival at 72 h (Figure 6, *P *< 0.001, log rank test). Survival of UF-CB treated animals from pneumococcal infection at 72 h in over 4 separate experiments was between 40% and 60%, with surviving animals remaining disease-free for several days after the experiment.

### Tissue infection

**Figure 7:**
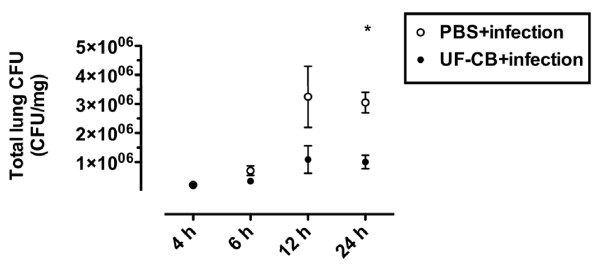
**Total lung tissue colony forming unit (CFU) counts in infected mice pre-treated with; i) 2× intranasal doses of sterile PBS, and ii) 2× intranasal doses of 500 μg ultrafine-carbon black (UF-CB)**. At 24 h post-instillation of *S pneumonaie*, there is a significant decrease in UF-CB treated animals. Data are described by mean ± SEM, n = 5 for each time point for each group. * *P *< 0.01 by unpaired t test.

**Figure 8:**
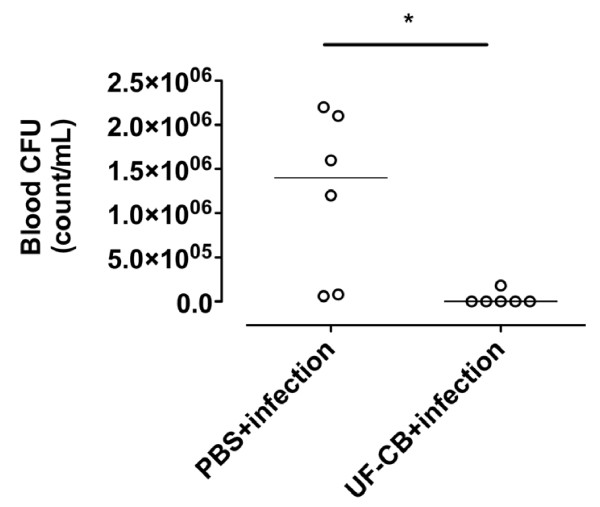
**Blood CFU counts in mice treated with either 2× doses of sterile PBS or 2× doses of 500 μg ultrafine-carbon black (UF-CB), then infected intranasally with pneumococci**. Blood CFU count was assessed at 24 h post-infection. n = 6, Bar represents mean, *P < 0.05 by unpaired t test.

**Figure 9:**
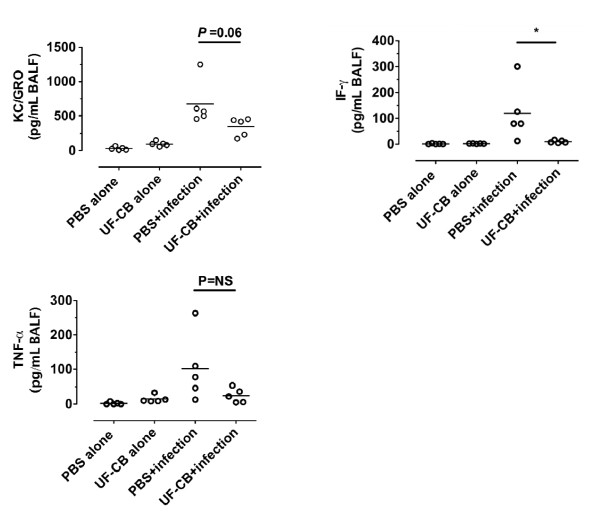
**Bronchoalveolar lavage (BAL) fluid cytokines**. BAL was done at the 24 hr post final exposure. Uninfected mice received; i) 2× doses of PBS alone, ii) 2× 500 μg ultrafine-carbon black (UF-CB) alone. Infected mice received either 2× doses of PBS, or 2× 500 μg ultrafine-carbon black (UF-CB), followed by instillation of pneumococci. Infection increased BAL fluid keratinocyte-derived chemokine/growth-related oncogene (KC/GRO), Tumor necrosis factor (TNF)-α, and interferon (IF)-γ (*P *< 0.05, PBS alone vs. PBS+infection). UF-CB pre-treatment resulted in lower levels of murine IL-8 and IF-γ. Bar represents mean, n = 5. * *P *< 0.05 by unpaired t test.

To determine whether increased survival after exposure to UF-CB was associated with decreased bacterial load in lung tissue, pneumococcal CFU counts were measured up to 24 h. Lung CFU counts were initially low in both PBS- and UF-CB-treated groups at 4 and 6 h after infection (*P *= NS, Figure 7). However by 12 h, lung CFU counts were lower in UF-CB treated animals, and by 24 h this decrease was significant (Figure 7, *P *< 0.01, n = 5). In a separate 24 h experiment, mice treated with UF-CB had significantly lower blood CFU counts (Figure 8, n = 6, P < 0.05 vs. PBS control). Exploratory analysis of BAL fluid cytokines was done in both uninfected and infected animals at 24 h. Treatment with UF-CB alone did not increase BAL fluid keratinocyte-derived chemokine/growth-related oncogene (KC/GRO), TNF-α or IF-γ (Figure 9, n = 5). Pneumococcal infection increased BAL cytokines at 24 h (*P *< 0.05 vs. uninfected). However, BAL fluid cytokines in infected mice treated with UF-CB tended be lower (Figure 9, KC/GRO; P = 0.06, TNF-α; P = NS, and IF-γ; P < 0.05).

## Discussion

This study was designed to assess the hypothesis that loading of AM with particles of ultrafine elemental carbon increases susceptibility to pneumococcal pneumonia. In conflict with the hypothesis, using an established animal model of pneumococcal pneumonia, we found that mice exposed to UF-CB were partially protected against the consequences of pneumococcal infection, with delayed morbidity and reduced mortality at 72 h - and no evidence of late-onset infection in the surviving animals.

This is the first report of a protective effect of carbonaceous PM on bacterial pneumonia. To date, increased susceptibility to bacterial infection has been reported after exposure to environmental PM. For example in rats, inhalation of aerosolised diesel exhaust particles (20 mg/m^3 ^for 4 h a day for 5 days) followed by *Listeria *infection, increased *Listeria *lung CFU counts [11]. In another example, a dose-dependent increase in *S. Aureus *CFU counts occurred in rabbits after inhalation of wood smoke for 1 h per day for 4 days [12]. One previous study has addressed the interaction between carbonaceous PM and susceptibility to pneumococcal pneumonia. Sigaud *et al *[13] exposed BALB/c mice to aerosolised interferon gamma, then instilled concentrated ambient particles intra-nasally, followed by infection with *S. pneumoniae*. In this model, all untreated mice survived and cleared instilled bacteria by 24 h. By contrast, mice exposed to concentrated ambient particles and interferon gamma, had increased BAL neutrophils and more severe pneumonia at 24 h. However, there are significant differences between our model and that of Sigaud *et al *[13], i.e. we used UF-CB as a surrogate for the carbonaceous fraction of biomass PM, and all untreated mice died from pneumococcal disease by 72 h.

Lung tissue injury, by damaging airway epithelial integrity would be expected to enhance susceptibility to bacterial infection. Indeed, we found changes in lung histology after UF-CB installation suggestive of mild tissue injury. However, UF-CB treatment resulted in consistently lower levels of pulmonary pneumococcal CFU counts after infection, and attenuated the induction of KC/GRO, and IF-γ. Since there was no difference in lung CFU counts immediately after instillation of bacteria (data not shown) and at 4 h and 6 h after infection in UF-CB and PBS-treated groups, the possibility that UF-CB treated mice had lower number of instilled bacteria is excluded. Compatible with the reduced lung CFU counts up to 24 h in UF-CB exposed mice, and lower pro-inflammatory cytokine levels, and fewer carbon-exposed animals developed pneumococcal bacteraemia at 24 h.

The mechanism whereby an acute does of UF-CB protects mice against pneumococcal morbidity and mortality is unclear. Increased anti-pneumococcal function in carbon-laden AM is unlikely, since loading of AM with UF-CB *in vitro *significantly impairs pneumococcal killing [6]. In the rat, instillation of endotoxin results in recruitment of neutrophils into the lung and protection against subsequent death from *Pseudomonas aeruginosa *pneumonia (0% vs. 54% in controls) at 24 h [14]. Similarly, we found in both BAL and lung tissue, that UF-CB induces a mild pulmonary neutrophilia. Endotoxin contamination of UF-CB is not a stimulus for the pulmonary neutrophilia observed in the present study, since particles were baked at high temperature for several hours before use. Previous animal studies have shown UF-CB alone, via oxidative stress, induce a low grade airway neutrophilia [15]. Increased urinary 8-oxodG in UF-CB-exposed mice in the present study is suggestive of increased pulmonary oxidative stress [16,17]. We therefore speculate that partial protection against pneumonia in UF-CB exposed mice is due to oxidative stress stimulating the recruitment of neutrophils into the airway.

There are several limitations to our data. First, human AM acquire inhaled carbon over several months. To achieve a comparable degree of AM carbon loading, requires a high acute dose of ultrafine carbon black (2 × 500 μg per animal). The effect of chronic loading of murine AM with repeated low doses of UF-CB *in vivo *on vulnerability to pneumococcal pneumonia remains to be determined. Second, we have used lethal dose of pneumococci which does not model the effect of inhaled carbon on pneumococcal colonisation of the upper airway. However, pneumococcal pneumonia is a leading cause of death in young children - and our results therefore model vulnerability to death in children with established infection. Third, biomass PM consists not only of carbon nanoparticle aggregates (modeled in this study), but also adsorbed compounds such as metals (which we have not modeled). The potential importance of adsorbed compounds is suggested by Zhou and Kobzik [7], who reported decreased internalisation of *S. pneumoniae *and killing by a murine macrophage cell line loaded with concentrated ambient particles *in vitro*, but no impairment of internalisation when cells were loaded with carbon black. Indeed, we have previously shown that adsorbed metals in dung-smoke PM_10 _contribute to the induction of oxidative stress in a cell-free system [18]. Future animal studies of susceptibility to infection should therefore focus on unprocessed biomass PM.

## Conclusion

We found no evidence to support our hypothesis that the epidemiological association between exposure to biomass smoke PM and susceptibility to pneumonia is due to high levels of carbon loading of AM *per se*. In female MF1 mice, pre-treatment with UF-CB results in lower lung and blood pneumococcal burden, and reduced morbidity and mortality. The mechanism for the protective effect is unknown, but may be due recruitment of low numbers of activated neutrophils into the lung prior to infection.

## Methods

### Animals and ultrafine carbon black

Female outbred MF1 mice (Harlan Olac, United Kingdom) aged 9 wk, were used for all the experiments. Animals were specific pathogen-free and provided with water and food pellets *ad libitum*. Ultrafine carbon black (UF-CB) (Printex 90, 14 nm diameter spherules, Degussa, Frankfurt, Germany) was a kind gift from Professor Ken Donaldson. UF-CB was baked at 240°C. for 12 h before use. Prior to instillation, UF-CB was added to sterile PBS to produce a 10 μg/μL suspension. The suspension was sonicated 5× for 30 s prior to use.

### Streptococcus pneumoniae

*S. pneumoniae *serotype 2 strain D39 was obtained from the National Collection of Type Cultures, London, UK. Pneumococci were cultured in Brian Heart Infusion (BHI) broth containing 20% (v/v) fetal calf serum or on blood agar base containing 5% v/v horse blood. Bacteria were confirmed as pneumococci with an optochin disk (Oxoid, Basingstoke, UK) and Gram staining. Before use in intranasal infections, pneumococci were passaged through the mouse peritoneum and collected from cardiac blood as described previously [17]. Briefly, the inoculum was injected intra-peritoneally into mice and the cardiac blood was collected form terminally anesthetised animals at 24 h. Pneumococci were harvested the next day by centrifugation, and resuspended in BHI containing 20% (v/v) fetal calf serum for 4 h at 37°C to an OD_500 _of 1.6. Bacterial viability count was assessed by counting colony forming units (CFU) on blood agar base plates with 5% (v/v) horse blood (Oxoid, Basingstoke, UK). Aliquots of bacteria were then stored at -80°C. for mice infection experiments.

### Infection protocol

To assess the effect of exposure to UF-CB on susceptibility to pneumococcal infection, animals first received either intranasal sterile PBS or intranasal UF-CB (500 μg UF-CB in 50 μL sterile PBS) on day 1. To instil fluid into the lower airway, animals were lightly anesthetized with 2.5% (v/v) fluothane (AstraZeneca, Macclesfield, UK) over oxygen (1.5 to 2 L/min), held by the back of the neck, and sterile PBS or UF-CB suspended in PBS dropped onto the nose. On day 4, intranasal instillation of either PBS or UF-CB was repeated (i.e. total of 1000 μg). On day 5, animals received either intranasal 50 μL sterile PBS or 1 × 10^6 ^colony forming units (CFU) *S. pneumoniae *in 50 μL sterile PBS [19]. Mice were regularly monitored for signs of disease for up to 72 h after infection, or until they became severely lethargic, at which point animals were humanely sacrificed. To assess tissue CFU, lungs were homogenized in 5 mL sterile PBS with a tissue homogenizer (Jencons, Leighton Buzzard, UK). Serial dilutions were performed up to 10^6 ^in using PBS as diluent. Dilutions of homogenates (20 μL) were placed onto blood agar and the number of CFU counted after overnight incubation at 37°C. Blood was also collected immediately post-mortem.

### Bronchoalveolar lavage of mice

Bronchoalveolar lavage was obtained from mice by instilling 4 × 500 μL sterile PBS through a 25-gauge needle into the lungs, via the trachea, followed by aspiration of BAL fluid into the syringe. BAL fluid was pooled form each animal and samples were kept on ice. BAL lavage fluid was cytocentrifuged (Shandon Instruments; Pittsburgh, PA), and cells stained with Diff-Quik (Dade Behring; Deerfield, IL, USA). The BAL fluid neutrophil differential count was obtained from randomly selected fields (light microscopy, oil immersion, ×100) from 300 leukocytes. Carbon loading of AM was determined in 300 randomly selected AM using a semi-quantitative scale. Lightly-laden AM had less than 10% cytoplasmic particle burden, moderately-laden cells had 10 to 50% of the cytoplasm occupied by particles, and heavily-laden cells had more than 50% of their cytoplasm containing carbon black.

### Bronchoalveolar lavage of humans

Sampling and BAL fluid processing of humans were done as previously described a biomass-smoke exposed population living in Blantyre, Malawi as a part of a previously published study into the association between solid fuel use and AM carbon-loading [8]. Sampling received ethical approval from the Research Ethics Committee of the Liverpool School of Tropical Medicine and the College of Medicine, University of Malawi.

### Bronchoalveolar lavage fluid cytokines

Exploratory analysis of BAL fluid cytokines was done using a 96-well multi-spot plate for mouse cytokines obtained from Meso Scale Discovery (MSD, Gaithersburg, USA). The assay was performed according to the manufacturer's instructions. The time of incubation for standard and samples was 1.5 h. The plate was read using a MSD SECTOR imager and data analyses was done using the discovery workbench software. Keratinocyte-derived chemokine/growth-related oncogene (KC/GRO) - the murine ortholog of human interleukin-8, tumor necrosis factor alpha (TNF-α) and interferon gamma (IF-γ) were defined *a priori *as the cytokines of interest.

### Urinary 8-oxodG assay

To collect urine, mice were placed in metabolism cages. Urine was pooled and frozen at -80°C. prior to analysis. For analysis of 8-oxo-deoxyGuanosine (8-oxodG), a 100 μL aliquot of mouse urine sample was diluted with 890 μL of HPLC grade water and spiked with 10 pmol of the stable isotope internal standard [^15^N_5_]8-oxodG (1 pmol/μL), which was synthesized as described previously [20]. Urine samples were then subjected to solid phase extraction using Oasis HLB columns (1 mL, 30 mg, Waters Ltd., Elstree UK), evaporated to dryness and redissolved in 50 μL of HPLC grade water The purified urine samples were subjected to liquid chromatography-electrospray ionization-tandem mass spectrometry (LC-ESI-MS/MS) with selected reaction monitoring (SRM) analysis by injecting a 10 μL aliquot of each sample in triplicate as described previously [21]. Selected reaction monitoring analysis was performed for the [M+H]^+ ^ion to oxidised base [B+H_2_]^+ ^transitions of 8-oxodG, *m/z *284 to 168 and the internal standard [^15^N_5_]8-oxodG, *m/z *289 to 173. The level of 8-oxodG in each urine sample was determined from the ratio of the peak area of 8-oxodG to that of the internal standard and normalized to the specific gravity which was determined using a Reichert TS 400 refractometer (Reichert Analytical Instruments, Depew, USA). The levels of 8-oxodG are expressed in pmol/mL relative to a specific gravity of 1.036 which is the average of all the samples.

### Lung histology

Whole lungs were fixed in formalin for 24 to 48 h and embedded in paraffin for histological sectioning. Sections were stained with hematoxylin and eosin and analysed under light microscopy (200 to 400x) by an experienced in veterinary pathologist blinded to exposure status.

#### Statistical analysis

Data are summarized as mean (standard error of the mean; SEM) and were analysed using GraphPad Prism version 5.03 (GraphPad Software Inc., La Jolla, CA). Survival results were validated by 4 separate experiments done at different times with n = 10 in each group. Comparison of survival was done by log rank test. Other data, except for the exploratory BAL cytokines data (n = 1 experiment), were validated by at least 3 separate experiments done at different time. Data were compared by the unpaired t test. A *P *value of <0.05 was considered significant.

## Abbreviations

AM: alveolar macrophage; BAL: bronchaoveloar lavage; CFU: colony forming unit; LC-ESI-MS/MS: liquid chromatography-electrospray ionization-tandem mass spectrometry; 8-oxoxdG: 8-oxo-deoxyGuanosine; PBS: phosphate buffered saline; PM: particulate matter; HPLC: high performance liquid chromatography; UF-CB: ultrafine carbon black

## Competing interests

The authors declare that they have no competing interests.

## Authors' contributions

JG designed the experiments and wrote the manuscript. JG and PA supervised the experiments. PA critically revised the original version. AT and VF did the mouse experiments. AT helped design experiments, and contributed in drafting the manuscript. FT and RS performed the analysis of 8-oxoxdG. JR and SG performed the human lavage study and contributed to drafting the manuscript. All authors read and approved the final manuscript.
